# Sensory Analysis and Consumer Research in New Product Development

**DOI:** 10.3390/foods10030582

**Published:** 2021-03-10

**Authors:** Claudia Ruiz-Capillas, Ana M. Herrero

**Affiliations:** Institute of Food Science, Technology and Nutrition (ICTAN-CSIC), José Antonio Novais 10, 28040 Madrid, Spain; ana.herrero@ictan.csic.es

**Keywords:** sensory analysis, sensory properties, consumer research, new product development, healthier products, food quality and safety, food design, quantitative descriptive analysis (QDA), check-all-that-apply (CATA), napping, flash profile (FP), temporal dominance of sensations (TDS)

Sensory analysis examines the properties (texture, flavor, taste, appearance, smell, etc.) of a product or food through the senses (sight, smell, taste, touch and hearing) of the panelists. This type of analysis has been used for centuries for the purpose of accepting or rejecting food products. Historically, it was considered a methodology that complements technological and microbiological safety when assessing the quality of food. However, its important evolution and impact in recent decades has placed it as one of the most important methodologies for innovation and application to ensure final product acceptance by consumers. Traditional sensory techniques, such as discriminatory, descriptive evaluations, preference and hedonic tests, which are still widely used today, have evolved into newer, faster and more complete techniques: check-all-that-apply (CATA), napping (N), flash profile (FP), temporal dominance of sensations (TDS), etc., together with an important and adequate statistical analysis. All of these techniques, with their advantages and disadvantages, are very useful in the development of new foods. However, it is not only sensory characteristics that determine the acceptance or success of a new product. Factors such as social aspects, the environment, nutritional knowledge, specific diets, emotions, health, the nature of the products, packaging, etc., also have a very important influence. New food product developers should take into account the attitudes and expectations of potential consumers. Consumers describe a product’s benefits by perceived intrinsic and extrinsic characteristics. For example, focus groups could be planned to identify different consumer expectations for new products. Properly measuring these factors and emotions will also have a very decisive influence on the success or failure of new product developments. A better understanding of the sensory experiences of potential consumers opens up a space and the inspiration for innovation. For all these reasons, sensory analysis, together with consumer research, is currently considered by the industry and researchers to be one of the most useful tools at the different stages of new product development, from design to commercialization, to improve the quality of products and to guarantee the success of innovation in market uptake among consumers. All these aspects are collected by Ruiz-Capillas et al. [1] in their review, which helps to better elucidate these techniques and enhance knowledge in this field, in order to facilitate the choice of the most appropriate aspect at the time of its application in the different stages of new product development, particularly regarding meat products.

Furthermore, this Special Issue (SI) also includes different sensory and consumer studies used for their application in the development of very different new products. Kalumbi et al. [2] evaluated consumer acceptability of new enriching maize-based stiff porridge with flour made from hydrothermally treated soybeans. This development could significantly contribute towards reducing the burden of energy–protein under-nutrition in populations in sub-Saharan Africa. On the other hand, Szymandera-Buszka et al. [3] employed consumer tests and sensory profiling to assess the impact of ethanol extracts of spices (lovage, marjoram, thyme, oregano, rosemary and basil) on the sensory quality of new pork meatballs and hamburgers. This work noted the usefulness of these techniques in the development of products with clean labeling, replacing synthetic preservatives with natural plant extracts. Tao and Cho [4] evaluated the sensory characteristics of Rebaudioside (Reb) A, D and M compared with sucrose, using a consumer panel, and explored the relationship between 6-n-Propylthiouracil (PROP) taster status (i.e., non-tasters, medium tasters, supertasters) and the perceived intensity of sweet and bitter tastes of the three steviol glycosides. Consumers were instructed to rate the sweetness and bitterness intensities of different solutions and a check-all-that-apply (CATA) question was used to evaluate the taste, which are considered to be more flexible and less time-consuming methodologies than traditional ones.

Other authors have analyzed the association between consumer perceptions of food quality and their acceptance of enhanced meat products and novel packaging [5]. This study was conducted using the Computer-Assisted Personal Interview (CAPI) method in a random group of 1009 respondents. The results suggested that educating consumers may improve their acceptance of product enhancement, as concerns about the addition of food preservatives may lead them to reject enhanced products. Biro et al. [6] also study sensory evaluation (CATA) together with technological parameters (color, hardness, etc.) to assess consumer acceptance of insects as food. These authors valued the acceptance or rejection of oat biscuits enriched with insect powders [*Acheta domesticus* (house cricket)]. An important part of this study was to discover how the insect content of the products affects the overall liking (OAL) and which attributes are the drivers of liking.

The potential to design natural tea-infused set yoghurt was also investigated using quantitative descriptive profile analysis and a consumer hedonic test together with technological properties (texture analysis, yield stress, physical stability and color) [7]. Three types of tea (*Camellia sinensis*)—black, green and oolong tea—as well as lemon balm (*Melissa officinalis* L.), were used to produce set yoghurt and compare this with plain yoghurt. Both types of yoghurt were also characterized by a high consumer willingness to buy. Principal component analysis (PCA) was used to analyze differences between samples and the correlation of selected variables.

Studies of different consumers from different countries have also been presented in this SI. Grasso and Jaworska [8] studied the presence of hybrid meat products in the UK market, extracted UK online consumer reviews on hybrid meat products and gathered preliminary consumer insights, utilizing the tools and techniques of corpus linguistics. These studies are of great importance since hybrid meat products could open up new business opportunities for the food industry and a greater diversity of products for consumers. Silva et al. [9] also carried out a preliminary inquiry with 155 consumers from Região de Lisboa and Vale do Tejo (Center of Portugal) to assess fish consumption, the applicability of fish product innovation and the importance of valorizing discarded fish (blue jack mackerel, black seabream, piper gurnard, etc.). Five products (black seabream ceviche, smoked blue jack mackerel pâté, dehydrated piper gurnard, fried boarfish and comber pastries) were developed and investigated for their sensory characteristics and consumer liking by hedonic tests by 90 consumers. The knowledge of consumers’ interests acquired with the data from the initial survey allowed for the development of new fish products, with the addition or substitution of the fish species under study through the reformulation of existing products that are familiar to the consumer. Głuchowski et al. [10] also used descriptive quantitative analyses and consumer tests to explore sensory characteristics, consumer liking of key attributes, their declared sensations and emotions, as well as consumers’ facial expressions when responding to the six dishes prepared using lemon or tomatoes and made in the traditional (classical), molecular and Note by Note (NbN) versions. Tests included a nine-point hedonic scale for degree of liking a dish, check-all-that-apply (CATA) for declared sensations and FaceReader for facial expressions. The influence of factors associated with consumer attitudes toward new food and willingness to try the dishes in the future were also determined. Such an approach was valuable in modifying features such as the taste, flavor and texture of dishes according to consumers’ points of view. The goal of the Kumar et al. [11] study was to highlight one strategic framework to find white spaces in the marketplace and then develop new snack texture concepts to fit the sensory concepts identified as white spaces. This paper shows one method of how new product concepts can be developed using such sensory science tools as product categorization, projective mapping and descriptive profiling. This research approach for novel and distinctive market opportunities displays an innovative, practical side of NPD research as a complement. The methodology produced in this study can be used by food product developers to explore new opportunities in the global marketplace.

Finally, Świąder and Marczewska [12] explored the current trends of using sensory evaluation in NPD in the food industry in countries that belong to the EIT Regional Innovation Scheme (RIS). A computer-assisted self-interviewing (CASI) technique for survey data collection was used. The research results showed that almost 70% of companies apply sensory evaluation methods in NPD and more so the bigger the company. Here, it was also noted that most companies prefer consumer (affective) tests to expert tests.

## Conclusions and Future Outlook

To summarize, the works collected in this Special Issue serve to offer an interesting contribution to the field and a better understanding of sensorial techniques and consumer research as a tool for innovation in new product development in order to satisfy the demands of consumers who increasingly seek new flavors, pleasure and fun and for companies to gain a better market position. Therefore, this SI is very useful for both present and future use for the different players involved in this kind of product development (industry, companies, researchers, scientists, marketing, merchandising, consumers, etc.). However, although these techniques have evolved substantially in recent years, the potential of using sensory evaluation methods is not yet fully exploited. A great future is predicted for them and a significant development is expected in the coming years. Finding new opportunities in food product development is a challenging assignment.
